# Traumatic brain injury and neurological stealth syndromes

**DOI:** 10.3389/fnins.2026.1879688

**Published:** 2026-07-15

**Authors:** Michael Hoffmann, Christian King, Edward Ross

**Affiliations:** 1 UCF College of Medicine, University of Central Florida, Orlando, FL, United States; 2 University of Central Florida, Orlando, FL, United States

**Keywords:** behavioral neurology, cognitive syndromes, FTD, superlative abilities, TBI

## Abstract

**Introduction:**

A traumatic brain injury (TBI) of mild or more severe degree affects approximately ½ of the global population at some stage of their life. Mild TBI occurs in 70–90%, with 30 and 50% having symptoms persisting for more than 6 months. Mild TBI presentations include cognitive, elementary neurological, neuropsychiatric, endocrine, autonomic, cardiac, and general medical entities, with many behavioral neurological syndromes flying under the radar.

**Aims:**

A retrospective examination of the cognitive and behavioral impairments in people with traumatic brain injury to evaluate the range of differing syndrome presentations, including hypofunction, hyperfunction and superla+ve brain function syndromes.

**Methodology:**

The Brainbeat Cognitive Registry was a prospectively designed observational registry that collected clinical, cognitive, behavioral, neurological, neuropsychiatric, laboratory, and radiographic data from people with cognitive and behavioral disorders.

**Results:**

In the registry (*n =* 73), of predominantly men (88%), with averages for age 55.1 years, BMI 28.9, education 15.1 years, and MOCA score 21.7. Migraine, olfactory impairment, depression, anxiety, and PTSD were all relatively commonly associated conditions. Relatively common disorders with more complex syndromes, including Diogenes syndrome, IEED, ADHD, field-dependent behavior, and hyperorality, the later on presenting as a human Klüver Bucy syndrome. Less common disorders included other higher cortical function disorders (17.1%), neuropsychiatric (10.5%), cortico-ponto- cerebellar pathway syndromes (10.5%), and visual radiation disorders (6.5%). The least common were chronotaraxis, schizophrenia, bipolar disorder, content-specific delusions, tremor, ataxia, astereopsis, and prosopagnosia. The majority of TBI patients presented with an overarching frontotemporal disorder (FTD) diagnosis (*n =* 68, 89.4%), with abnormal FRSBE scores for one or more entities of abulia, disinhibition, and executive dysfunction. Frontal Behavioral Inventory scores were abnormal in 86%. The most common neurological sub-syndrome was Geschwind-Gastaut syndrome (*n =* 49, 62.8%). A category of patients demonstrating superlative abilities (*n =* 9), including visual art, musical, literary, architectural brilliance, and precognition, all attributed to right hemisphere hyperfunction, was also identified.

**Conclusion:**

Post-TBI frontotemporal disorders are common. Deconstructing the overarching FTD diagnosis into multiple subsyndromes is clinically useful, revealing hypofunction syndromes, hyperfunction, and superlative function syndromes. The range of neurological stealth syndromes as part of the post-TBI range of maladies may facilitate a more targeted, precision management approach.

## Introduction

A traumatic brain injury (TBI) of mild or more severe degree affects approximately ½ of the global population at some stage of their life. About 13.8 million in the USA sustain mild TBI, 55.9 million globally, and adults ≥ 65 years accounted for 43.9% of all TBI-related hospitalizations (falls) and 38.4% of TBI-related deaths (US, 2017). Based on the level of consciousness, 70–90% is mild TBI, and it has been presumed that most recover relatively quickly. However, mild TBI, in itself, is a misnomer, as between 30 and 50% have symptoms persisting for>6 months, causing disruptions to employment and relationships, with an estimated cost of approximately 17 billion annually in the USA (2017) (Maas et al., 2017; Dewan et al., 2018).

Mild TBI has wide-ranging presentations, recently highlighted in a meta-analysis involving over 31 million participants (Botchway et al., 2024). An increasing array of symptoms and signs continues to be recorded in people who have incurred a TBI, spanning the domains of cognitive, behavioral, neurological, neuropsychiatric, elementary neurological, endocrine, autonomic, cardiac (Takotsubo cardiomyopathy), as well as a range of general medical entities (Mikolić et al., 2023; Gruhl et al., 2022). Furthermore, there is increasing evidence for a multitude of behavioral neurological syndromes flying under the radar, as reported in a recent Veterans Health Administration-based retrospective review (Hoffmann et al., 2024). This is unsurprising given the complex TBI pathophysiology, including neurotransmitter, vascular, and metabolic perturbations, all of which contribute to these syndromes. Mild TBI diagnosis is both challenging and an accurate diagnosis, critical for implementing appropriate clinical care. Another large VHA study highlighted the relatively high rate of missed diagnoses by current screening protocols (Walker et al., 2024). Even more concerning, some neuropsychologists have opined that there is indeed a fundamental problem in the type of assessments applied to TBI patients that contrasts with the significant neuroimaging advances and current-day pathophysiological understanding of the condition. In addition, the outdated prediction of a rapid recovery in mild TBI also needs to be discarded. These concerns attracted an influential, four-part manuscript series by neuropsychologists, wherein they delineate these considerations (Bigler et al., 2024). To miss treatable syndromes associated with TBI is at best a missed opportunity for meaningful management or treatment and at worst a tragedy for the person and their family. Not infrequently, the cognitive and behavioral manifestations of TBI may present with a frontotemporal syndrome (FTS) (Wang et al., 2015; Deutsch et al., 2014). Frontotemporal syndromes may be regarded as a generic presentation of a common group of neurodegenerative diseases as well as a myriad other etiologies. This must be distinguished from a diagnosis of frontotemporal degeneration and frontotemporal dementia (FTD) when the activities of daily living evaluations supported the dementia category, with frontotemporal degeneration a more generic term in the absence of supporting genetic markers of frontotemporal dementia.

A previous report by the authors delineated a number of less common, but nevertheless significant sub-syndromes subsumed under the umbrella of FTS (Hoffmann et al., 2024). FTS are amongst the most common brain neurodegenerative disorders, and their relatively covert, frequently subtle presentations and diverse etiologies pose major challenges in diagnosis and treatment. These include the mild FTD phenocopy syndrome that may remain pauci-symptomatic for decades with only very slow progression (Overvlieta et al., 202).

The range of FTS themselves continues to expand in their mode of syndromic presentations, as well as etiologies, with over a dozen reported other than frank frontotemporal dementias (Hoffmann et al., 2024). In addition to displaying a cognitive-behavioral dissonance, hemispheric hyperfunction and other superlative syndromes consequent to TBI occur and have seldom been reported post brain injury (Gesen et al., 2021). In recent publications by the authors, as well as others, TBI in particular was associated with a relatively high frequency of FTS sub-syndromes, notably the Geschwind-Gastaut syndrome (GGS), the human Klüver Bucy syndrome (KBS), involuntary emotional expression disorder, field-dependent behavior and delusional misidentification syndromes post TBI (Hoffmann et al., 2024; Clay et al., 2019; Toomukuntla et al., 2025).

As a follow-up study, a prospectively designed TBI registry was developed to facilitate the elicitation of these more covert syndromes that are rarely volunteered by the patient or family and generally require elicitation by direct questioning and testing. These so-called neurological stealth syndromes may take the form of hypofunction, hyperfunction, or manifest with superlative abilities. Neurological Stealth syndromes (such as GGS, KBS) were considered an appropriate term insofar as several of the syndromes evade conventional neuropsychological testing, as specific behavioral neurological testing is required to ascertain these. This paragraph has been included in the manuscript.

The well-established theory of diaschisis that may manifest with clinical symptoms and signs, after many different kinds of brain afflictions, has been well supported by functional neuroimaging such as diffusion tensor imaging and metabolic PET imaging (Mollica et al., 2025; Thiebaut de Schotten et al., 2020). Such brain alterations, referred to as the disconnectome, may well be in a position to explain the more covert and disparate neurological stealth syndromes but this remains conjectural. The frequency of such various and less common cognitive and behavioral neurological deficits, to our knowledge, has not been addressed in a large TBI population.

## Aims

To retrospectively examine the cognitive and behavioral impairments in people with traumatic brain injury to garner the prevalence estimation of differing syndrome presentations using a dedicated, cognitive, and behavioral neurological registry.

## Methodology

The Brainbeat Cognitive Registry was a prospectively designed observational registry that collected clinical, cognitive, behavioral, neurological, neuropsychiatric, laboratory, and radiographic data from people with cognitive and behavioral disorders over 3 years (April 2021–March 2024). The study and design, and target populations included consecutive referrals to the cognitive neurology clinic from community medical practices, neurological practices, and regional VA Medical Centers. Subsequent retrospective analysis was performed sanctioned by IRB and ethics approval on March 4, 2024, WCG IRB Work Order #1–1745468-1, registered as Brainbeat Cognitive Registry of cognitive and behavioral neurological syndromes with a specific focus on the TBI population. The WCG IRB approval specifically stipulated the strict de-identification of all data prior to any reporting or publications emanating from this registry.

The rationale of the current study design was based on prevalence estimation and influenced by the first author’s previous findings of extensive and wide-ranging cognitive and behavioral sequelae, with the grouping of syndromes based on the frequencies of clinical presentation, from common, relatively common, less common, and the rarer superlative syndromes (Hoffmann et al., 2024). Of specific interest, there was a high frequency of frontotemporal syndromes (FTS) in the TBI populations for which the diagnostic tools included the DAPHNE criteria (a six-domain measurement of disinhibition, apathy, perseverations, hyperorality, personal neglect, and loss of empathy) (Boutoleau-Bretonnière et al., 2015) and the Frontal Behavioral Inventory (FBI) score (Kertesz et al., 1997). FTS syndromes were delineated into five clinical 5 subtypes: behavioral, semantic aphasia, progressive non-fluent aphasia, and a miscellaneous group including corticobasal ganglionic, progressive supranuclear palsy (PSP), and amyotrophic lateral sclerosis (ALS). FTS is distinguished from a diagnosis of frontotemporal degeneration and frontotemporal dementia when the activities of daily living evaluations supported the dementia category, with frontotemporal degeneration a more generic term in the absence of supporting genetic markers of frontotemporal dementia. All patients were seen by a cognitive and behavioral neurologist as part of the initial and subsequent clinical assessments. A comprehensive cognitive and behavioral neurological screening tool with pre-defined syndromes, according to standard definitions, was used to guide initial diagnosis, published elsewhere (Hoffmann et al., 2009). Demographic, cardiovascular risk factors, cognitive, behavioral, neurological, and neuroimaging data were collected, allowing the diagnosis of presumed etiological categories to be made for those with a diagnosis of FTS. Cognitive and behavioral evaluations included a cognitive screening with the MOCA 5-min version test (Nasreddine et al., 2005; Wong et al., 2015), the Boston Naming Test (BNT), abbreviated version (Kaplan et al., 2001), and behavioral testing with specific frontal behavioral testing with the Frontal Systems Behavioral Evaluation (FRSBE) (Grace and Malloy, 2001), family rating as opposed to self-rating used due to reported neurobehavioral dissonance between the two (Leathem et al., 1998). Anterior temporal lobe tests included the modified (yes/no answers instead of 0–5 grading system) Bear-Fedio Inventory (BFI) (Bear and Fedio, 1977; Trimble and Freeman, 2006), which included 18 different syndromes answered jointly by the patient and significant other that were used to diagnose GGS, 9 of which were used to diagnose GGS in 5 major categories. Because of the relatively high incidence of GGS in TBI (Hoffmann et al., 2024; Trimble and Freeman, 2006), a strict definition of GBS with 4 out of the 5 core features was required to diagnose GGS, namely:

1. *Viscous personality*: considered to be the central component of GGS, that included circumstantiality, interpersonal viscosity, tendency to repetition, prolongation of personal encounters, loquacious, hyper-narrative, overinclusive verbal discourse, excessive detail of information, and pedantic.
2. *Hypergraphia:* excessive writing, including diaries, autobiographies, and random notes
3. *Intensified mental life:* deepening of emotions, hyper-moralism, sense of personal destiny, philosophical - nascent metaphysical or moral speculations, cosmological theories, dependence, at the hands of fate, cosmic helplessness
4. *Religiosity:* multiple conversions, deep religious beliefs, mystical states
5. *Altered sexual interest:* hyposexuality, hypersexuality, gender dysphoria, transvestism

The Human Klüver Bucy syndrome required evidence of a minimum of 3 components of the following: (i) visual agnosia, (ii) loss of anger, fear responses with placidity or flattened affect, (iii) altered sexual activity or orientation, (iv) hyperorality, bulimia or insatiable appetite, and (v) hypermetamorphosis (compulsion to manipulate objects in the immediate environment, also termed utilization behavior) (Lilly et al., 1983; Clay et al., 2019).

Delusional misidentification syndrome (DMIS) was diagnosed if a person incorrectly identifies or duplicates persons, places, objects, or even events, which may be learned by self-report or substantiated by family members or friends (Devinsky, 2009). Although a number of different DMIS’s have been reported, for this study, only 3 more common types were recorded, including:

1. Capgras syndrome, which is the belief by the person that a familiar individual or even the person themselves has been replaced by an imposter (hypo-identification)
2. Fregoli’s syndrome, which is the belief that an individual familiar to the person is actually impersonating and is presenting themselves as a stranger (hyper-identification)
3. Intermetamorphosis, which refers to two people, both familiar to the person, who have interchanged identities with one another.

Involuntary emotional expression disorder (IEED), items 2 and 6 of the FRSBE test were used and were graded on a 5-point Likert scale, and if ≥3 was used as a positive diagnosis. This question delineated a syndrome characterized by spontaneous outbursts of crying, laughing, or both, occurring contextually inappropriately (Grace and Malloy, 2001; Cummings, 2007). Diogenes syndrome (personal neglect) diagnosis made if item number 11 of FRSBE test, a 5-point Likert scale, and if ≥3 was used as a positive diagnosis, or if item 6 of the FBI test was used if rated a 2 or 3 on a scale of 0–3 (Grace and Malloy, 2001). Field-dependent behavior: Environmental autonomy (imitation and utilization behavior). Imitation behavior. Maintaining eye contact, the examiner pats the side of the face and then claps the hands without suggesting the patient follow suit. Utilization behavior: Place three objects in front of the patient: a key, a cell phone, and a pen (Hoffmann et al., 2009). ADHD inventory diagnosis was made if item numbers 4 and 11 of the FRSBE test 5-point Likert scale, and if ≥3 was used as a positive diagnosis, and DSM-V (Grace and Malloy, 2001; DSM-5-TR, 2022). Depression, anxiety, and PTSD diagnoses were made in accordance with the DSM-5 (DSM-5-TR, 2022).

Neuroimaging with multimodality MRI imaging sequences (3 Tesla) MRI (T1, T2), fluid attenuation inversion recovery (FLAIR), and diffusion weighted imaging (DWI) was performed in all patients. Computerized tomography scans were used when MRI scans were contraindicated and indicated, and FDG, metabolic, positron, emission tomography (PET) scans were exclusively requested in selected patients, for diagnostic reasons when there was a clinical syndrome-MRI mismatch. PET scan neurodiagnostic readers were not blinded.

Routine cognitive impairment and dementia related tests were performed, and genetic testing (C9orf72, GRN, and MAPT) was not performed in the absence of a family history of FTD. Lack of utility, genetic counseling (if positive), patient preference, and cost factors are factored into the decision-making. FTD clinical subtypes were classified into the standard behavioral variant, semantic aphasia, non-fluent aphasia, cortico-basal degeneration variant, progressive supranuclear palsy variant, FTD, and amyotrophic sclerosis variant in accordance with the currently accepted classification (Murley et al., 2020). Etiological entities were based on clinical history, cognitive, behavioral, laboratory, and imaging analyses and categorized as:

1. Traumatic Brain Injury: Centers for Disease Control and ICD-10 criteria for mild and moderate TBI (Centers for Disease Control and Prevention, National Center for Injury Prevention and Control, 2015).
2. Frontotemporal lobe degenerations and dementias (Boutoleau-Bretonnière et al., 2015; Montenigro et al., 2014).
3. Screening test, FBI test (if the score is ≥24) (Kertesz et al., 1997).

## Statistical analyses

This study used linear probability models to examine the associations between syndrome diagnosis and principal tests. The models accounted for gender, age, years of education, and body mass index (BMI). While our analysis is mainly exploratory, a correction procedure was deemed unnecessary, and we applied the Benjamini-Hochberg correction. Although logistic regression is usually preferred for binary outcomes due to its theoretical consistency with bounded probability outcomes, the LPM was selected for a few reasons applicable to our relatively small dataset (*n =* 73). First, maximum likelihood estimation in logistic regression is known to be unstable in small samples with low-prevalence or high-prevalence outcomes, producing inflated standard errors, non-convergence, or complete separation. In this dataset, some models produced one or several of these issues. Second, marginal effects from LPM models are numerically close to logistic regression marginal effects for outcomes not near the distribution extremes, and the standard errors remain well-behaved. For the models where logistic models converged, we estimated the marginal effects, and they were consistent with the coefficients from LPM. The main potential limitation of LPM is that the model does not constrain predicted probability between 0 and 1, and assumes a constant marginal effect across the predictors, which is unlikely to hold at extreme values in the distribution. However, given the moderate prevalence of most outcomes in our analytical sample and the fact that the estimates between LPM and marginal effects from logistic regression were similar, this limitation appears to be less of a concern. Small samples can make maximum likelihood estimation in logistic regression unstable, which might lead to large standard errors, convergence issues, or inflated coefficient magnitudes. A significance level of *p*-values below 0.05 was chosen. Statistical analyses were performed using Stata Version 16 MP (Stata Corp, College Station, TX, USA) for data management and data analysis.

## Results

In the registry (*n =* 73), of predominantly men (88%), relatively young to middle aged (55.1 years), mildly overweight (BMI 28.9), education (15.1 years) as a group, the average MOCA score was 21.7 (Table 1). Migraine, elementary neurological syndromes such as olfactory impairment, and neuropsychiatric syndromes such as depression, anxiety, and PTSD were all relatively commonly associated conditions, with depression (60;78.9%) and migraine (56;73.6%), with migraine days/month 5.78 SD, with 6.22 being the most common. Other relatively common and less common elementary behavioral, neurological, neuropsychiatric, and elementary neurological disorders are depicted in Table 2. TBI patients presented with an overarching FTD diagnosis (*n =* 68, 89.4%) as diagnosed by abnormal Daphne scores as a primary diagnostic tool for FTD diagnosis. All patients with a FTD diagnosis had abnormal FRSBE scores for one or more of the 3 principal frontal presentations, namely abulia, disinhibition, and executive dysfunction, and FBI scores were abnormal in 86% (mean 33.6).

**Table 1:** Demographic and clinical characteristics of the registry sample (*n =* 73).

Variable	Mean/*n*	SD/%	Min	Max
Continuous variables				
Age (years)	55.2	14.0	29	88
Education (years)	15.1	2.3	10	22
Body Mass Index (kg/m^2^)	29.0	4.7	20	42
Sex, *n* (%)				
Male	64	(87.7%)	–	–
Female	9	(12.3%)	–	–
Handedness, *n* (%)				
Right-handed	66	(91.8%)	–	–
Left-handed	4	(5.5%)	–	–
Ambidextrous	3	(4.1%)	–	–

SD, standard deviation. Percentages may not sum to 100 due to rounding. Handedness *n* values estimated from registry proportions × 73.

**Table 2:** Frequency of neurological syndromes and disorders in the registry sample (*n =* 73).

Syndrome/Disorder (*n =* 73)	*n*	%
Common disorders		
Frontotemporal disorders	68	93.2
Depression	60	82.2
Migraine	56	76.7
Anxiety	55	75.3
Geschwind-Gastaut syndrome (GGS)	49	67.1
PTSD	47	64.4
Relatively common disorders		
Olfactory/taste impairment	34	46.6
Hyposmia, hyperosmia, or dysosmia	18	
Dysgeusia	16	
Diogenes syndrome	30	41.1
IEED	27	37.0
ADHD	24	32.9
Hyperorality/Human Klüver-Bucy syndrome	12	16.4
Field-dependent behavior	10	13.7
Less common disorders		
Higher cortical function disorders	13	17.8
Seizures (generalized or partial)	9	
Frontal lobe seizures	2	
Chronotaraxis	1	
Charcot-Wilbrand syndrome	1	
Neuropsychiatric disorders	8	11.0
Bipolar disorder	5	
Psychosis spectrum/schizophrenia	1	
Tourette’s syndrome	1	
Delusional misidentification syndrome	1	
Cortico-ponto-cerebellar pathway syndromes	8	11.0
Tremor	4	
Ataxia	4	
Visual pathway disorders	5	6.8
Astereopsis	2	
Oscillopsia	1	
Prosopagnosia	1	
Vertical diplopia	1	
Superlative or hyperfunction syndromes		
Hyperfunction or superlative abilities (total)	9	12.3
Prodigious memory	1	
Visual art	2	
Musicality	1	
Stand-up comedy	1	
Poetry/hypergraphia	1	
Multilingualism (newly acquired, fluent in 5 languages post-TBI)	1	
Architectural creativity	1	
AI-aided language and musical rendition	1	
Premonitions/clairvoyance	1	

The most common associated behavioral neurological sub-syndrome was Geschwind-Gastaut syndrome (GGS) (*n =* 49, 62.8%). Factors that had a statistically significant association with GGS included the FBI score, the Bear Fedio score, and the Boston Naming Test score, noted in Table 3. The Bear Fedio Inventory comprised 18 questions, 9 of which were used to diagnose GGS in 5 major categories, with the GGS questionnaire subcomponent frequencies noted in Table 4. A higher Bear Fedio score was associated with a greater probability not only of GGS but also of the individual components of hypergraphia, circumstantiality, emotionality, hyper moralism, obsession, personal destiny, philosophical musings, hyper religiosity, and sexual alteration.

**Table 3:** Linear regression model of the associations between syndrome diagnosis and principal tests.

Key independent variable	MOCA score	Boston naming	FBI Score	FRSBE A	FRSBE D	FRSBE E	FRSBE T	Bear Fedio	MRI brain	Daphne
Outcome		Test								FTD
FRS IEED	0.008	−0.011	0.001	−0.001	0.018**	0.005	−0.007	0.008	0.112	0.090
FRSB ADHD	−0.006	−0.012	0.014*	0.008	0.023**	0.016	−0.039*	0.021	−0.261	0.080
FBI Diogenes	0.026	−0.046	0.011	0.006	0.006	0.002	−0.010	0.003	−0.059	−0.199
DMIS	−0.004	0.009	0.000	−0.002	−0.001	−0.003	0.006	0.002	−0.006	−0.141*
FBI IM/UB	0.024	−0.062*	−0.003	0.000	0.004	−0.011	0.012	−0.011	0.021	−0.165*
GSS	0.003	0.055*	−0.013*	−0.002	0.004	0.003	0.001	0.088**	−0.081	−0.124
KBS	−0.005	−0.030	0.008	−0.006	−0.004	0.002	0.008	0.016	0.070	−0.070
Hypergraphia	0.028	−0.031	−0.004	−0.009	−0.009	−0.015	0.031	0.055**	−0.097	−0.128
Circumstantiality	−0.010	0.012	−0.004	0.004	0.001	0.007	−0.009	0.073**	−0.001	0.096
Viscosity	0.023	−0.009	−0.016*	−0.007	−0.009	−0.002	0.020	0.075**	−0.055	−0.369*
Emotionality	−0.008	−0.025	−0.010	0.011	0.016*	0.014	−0.035	0.068**	0.012	−0.007
Hyper moralism	−0.025	0.076*	0.011	−0.006	−0.001	−0.004	0.009	0.033*	−0.094	0.232
Obsession	−0.002	−0.020	−0.006	−0.008	−0.002	−0.014	0.024	0.071**	0.027	−0.140
Personal destiny	0.037	−0.039	−0.010	−0.004	0.004	0.008	−0.003	0.043*	0.081	−0.045
Philosophical	−0.016	−0.016	0.002	−0.003	−0.003	0.007	0.006	0.063**	0.028	0.104
Hyper religious	0.051**	−0.014	−0.012	0.004	0.008	0.020	−0.017	0.038**	0.193	−0.355*
Sexual alteration	0.012	0.009	−0.006	0.010	−0.002	−0.001	−0.007	0.079**	−0.023	0.331
Depression	−0.003	−0.002	0.000	0.012*	0.014*	0.015	−0.038*	0.011	0.153	0.282
Anxiety	−0.009	0.035	−0.001	0.016*	0.023**	0.028*	−0.058**	0.015	0.038	0.243
PTSD	0.019	−0.023	−0.006	0.010	0.014	0.017	−0.032	−0.008	0.299	0.210
Migraine	−0.005	0.070	0.003	0.013	0.018**	0.033**	−0.054**	0.006	0.062	0.181
Migraine per month										
Number of patients	73	73	73	73	73	73	73	73	73	73

FRSBE A (apathy/abulia), FRSBE D (disinhibition), FRSBE E (executive function), FRSBE T (total score). **p < 0.01, *p < 0.05. All models account for gender, education, age of patient, and body mass index.

**Table 4:** Geschwind-Gastaut syndrome (GGS) questionnaire subcomponent frequencies (*n =* 73).

GGS Subcomponent (*n =* 73)	*n*	%
Emotionality	46	63.0
Sexual alteration	39	53.4
Circumstantiality	36	49.3
Viscosity	36	49.3
Personal destiny	29	39.7
Philosophical increase	26	35.6
Hyper-religiosity	25	34.2
Hyper-moralism	21	28.8
Hypergraphia	18	24.7

% = percentage of total registry sample (*n =* 73). GGS was diagnosed in 49 of 73 patients (67.1%). Subcomponent frequencies reflect all registry patients meeting criteria for each feature, ordered by frequency of occurrence.

Other relatively common disorders included olfactory/taste impairments with more complex syndromes including, Diogenes syndrome, IEED, ADHD, hyperorality, often presenting as a human Klüver Bucy syndrome and field dependent behavior. Much less common disorders characterized into other higher cortical function (17.1%), neuropsychiatric (10.5%), cortico-ponto-cerebellar pathway syndromes (10.5%), and visual radiation disorders (6.5%), were notable for their diverse presentations yet important to identify to allow both precise diagnosis and targeted therapy where appropriate. For example, chronotaraxis, schizophrenia, bipolar disorder, content-specific delusions, tremor, ataxia, astereopsis, and prosopagnosia are all important for both the patient, caregiver, and family members to be aware of to guide care and rehabilitation (Table 2). Syndrome correlations, associated with a FTD diagnosis are depicted in Table 3. For example, a FTD diagnosis, as per Daphne criteria, is associated with a lower probability of DMIS, FBI IM/UB, and hyper religiosity. A higher FRSBE A score was associated with a lower probability of FTD Daphne diagnosis, and a higher FRSBE T score was associated with a greater probability of FTD Daphne diagnosis.

Stealth syndromes,” hyperfunction,” or “superlative abilities” were terms used to highlight important syndromes that so-called fly under the radar and connotes a degree of vigilance that needs to be emphasized to clinicians who literally have to contend with many dozens of presenting syndromes in the context of TBI. The term hyperfunction was used to describe these increased abilities of different kinds, aptitudes, or even newfound talents that have frequently been described after a number of brain injuries or conditions. The category of patients demonstrating superlative abilities (*n =* 9), comprising hyperfunction type of abilities, including visual art, musical, literary, architectural brilliance, and precognition, all attributed to right hemisphere hyperfunction, was also identified and noted in Table 2. The term hyperfunction was used to describe these increased abilities of different kinds, aptitudes, or even newfound talents that have frequently been described after a number of brain injuries or conditions. Other TBI studies have even documented savant type abilities and appearances, even though in the current studies, no obvious savant syndromes were recognized in the current study. A number of so-called psychic abilities were reported by several patients. A brief classification of such abilities has been described and includes precognition, extrasensory perceptions, telepathy, clairvoyance, and telekinesis. One person in particular described a plausible event that was considered clairvoyance, explicitly is a self-report only and clearly needs to remain conjectural. This will be published in more detail with accompanying images and supportive data in a subsequent manuscript. The claims by those patients who reported superlative abilities were at times by self-report only, and in such instances may be categorized as behavioral phenotype descriptors, devoid of definitive proof of enhanced capacity. However, in several instances, there was clear-cut documentation for those reporting superlative visual artistry poetic, extraordinary multilinguistic abilities, architectural resplendence, and artificial intelligence-aided new language publication.

Neuroimaging: All patients had undergone MR imaging, which was abnormal in 84.6%. The abnormalities included most commonly diffuse axonal injury, followed by subcortical white matter lesions, subcortical gray matter lesions, cortical contusions, and evidence of brainstem injury. As all patients were seen in the chronic phase of their TBI, no acute or subacute sequelae of trauma injury, such as subdural or epidural hematoma or intracranial hemorrhage, were discerned. In 23 patients, PET brain scanning was performed primarily for diagnostic reasons and was abnormal in 78.2%.

## Discussion

**Figure 1:**
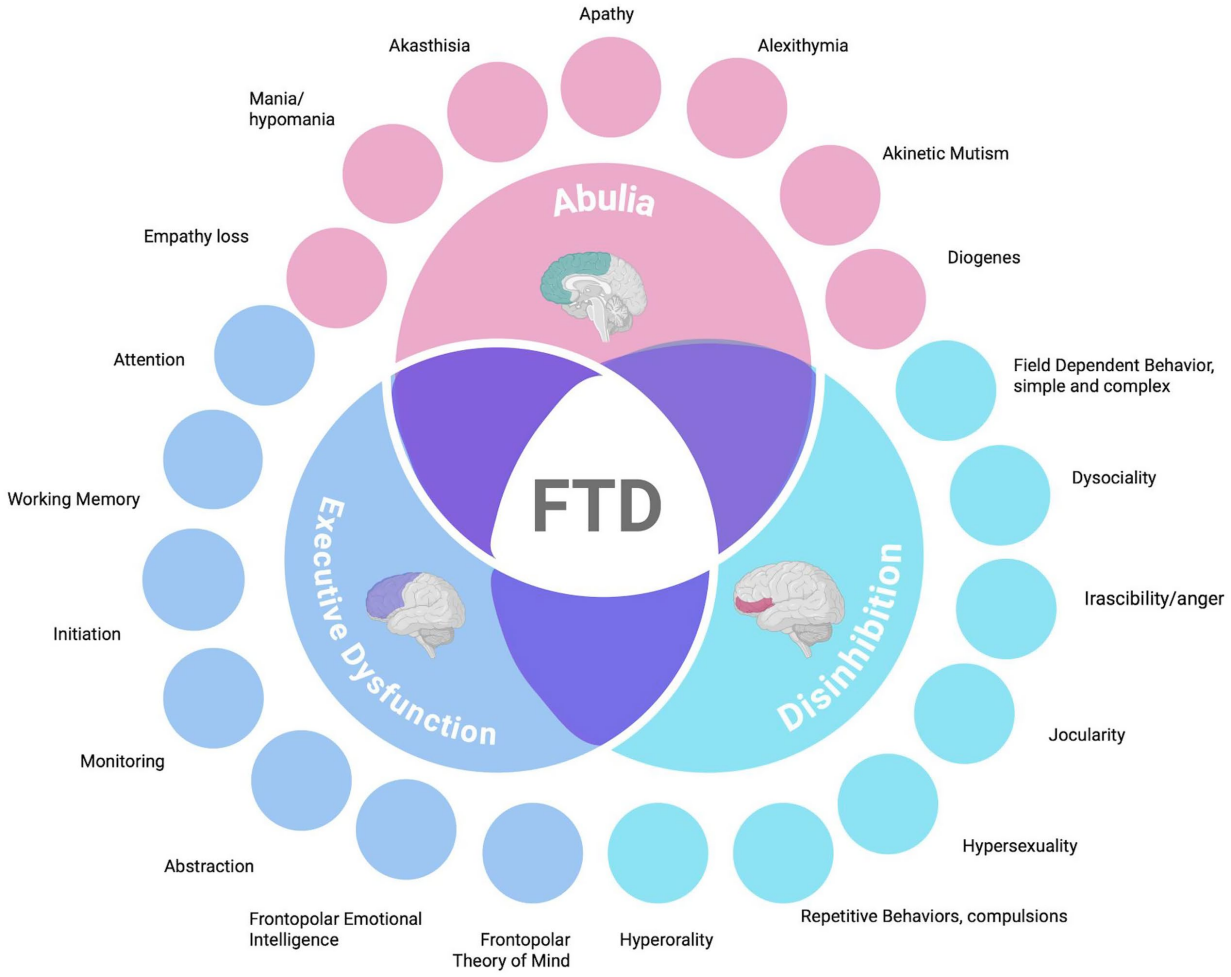
The three principal frontal Lobe syndromes, competencies and sub-syndromes that may oocur in frontotemporal disorders.

In the months to years after a traumatic brain injury, an extensive array of cognitive, behavioral, neuropsychiatric, and more elementary neurological syndromes may transpire. Frontotemporal syndromes in particular are relatively frequent, which is perhaps not surprising in view of the predilection of inferior frontal lobe and anterior temporal lobe injury reported in the neurosurgical, post-mortem neuropathological literature. In a recent clinical study of young soccer players with repetitive head injury, the orbitofrontal gray-white matter interface (GWI) was notable as a specific site of trauma associated with impaired cognitive performance due to GWI microstructural disruption (Song et al., 2025). Another study of relatively young footballers noted the onset of chronic traumatic encephalopathy diagnosed postmortem in the dorsolateral prefrontal cortex (Butler et al., 2025). Review of Figure 1 underscores the extensive subsyndromes that may emanate from the 3 principal frontal network domains, namely executive function, abulia (when conation is impaired), and the failure of inhibition resulting in disinhibition syndromes. Failure of inhibitory control is a frequent and fundamental derangement in people with frontotemporal disorders, which has been correlated with pervasive ventromedial and orbitofrontal prefrontal atrophy. The range of clinical tools measuring inhibitory assessment remains very limited and also relies heavily on caregiver input through specific questionnaires (O’Callaghan et al., 2013). Because of reliance on language and semantics, experimental tools such as the Flanker test used in the recently released, computerized Tabcat test may improve inhibitory function diagnostic capability for FTD as well (Njamnshi et al., 2025).

The GGS in particular was a relatively frequent diagnosis if the 5-item GGS criteria were applied, derived from the Bear Fedio Inventory. GGS has been previously reported both in association with FTD as well as TBI, mostly as case series and single case reports (Hoffmann et al., 2024; Postiglione et al., 2008; Veronelli et al., 2017). The critical role of delineating behavioral sub-phenotypes, that included GGS, in FTD and Alzheimer’s, for example, has recently been highlighted (Roy et al., 2023). First described in association with epilepsy, GGS has also been described in association with limbic encephalitis, brain tumors, and stroke (Benhamou et al., 2018; Salandra et al., 2022; Hoffmann, 2008). With regards to the underlying neurobiology of these syndromes, the phenomenon of the possible, relative dominance of left hemisphere language circuitry (referred to as the tyranny of the left hemisphere by some) being mitigated by lesions such as stroke or TBI may lead be related to its emergence due to Injury of the right hemisphere has been corroborated with the emergence of GGS, a kind of hyperfunction, in this instance manifesting with a preponderance of loquacity (hyper narration), excessive writing (hypergraphia), prodigious reading, excessive record, philosophical musings or philosophical concerns and hyper religiosity amongst other attributes. In support of this premise, the current reported literature supports right hemisphere lesions having a propensity to precipitate the Geschwind-Gastaut syndrome. The GGS syndrome definitions used have been the most rigorous to date, as per the available English literature. Nevertheless, it has become clear that behavioral neurological, and neuropsychiatric syndromes overlap substantially with the current diagnostic tools employed. Schizophrenia overlaps with bvFTD, depression, anxiety, bipolar disease, and GGS. Hence, overlap certainly applies to the manifold syndromes in this study. The specific tests for each have been delineated more clearly in the newly added Appendix 2. Similarly, left hemisphere lesions or relative right hemisphere hyperfunction post-TBI may precipitate artistic or musical abilities, creativity, and rarely savant syndromes.

**Figure 2:**
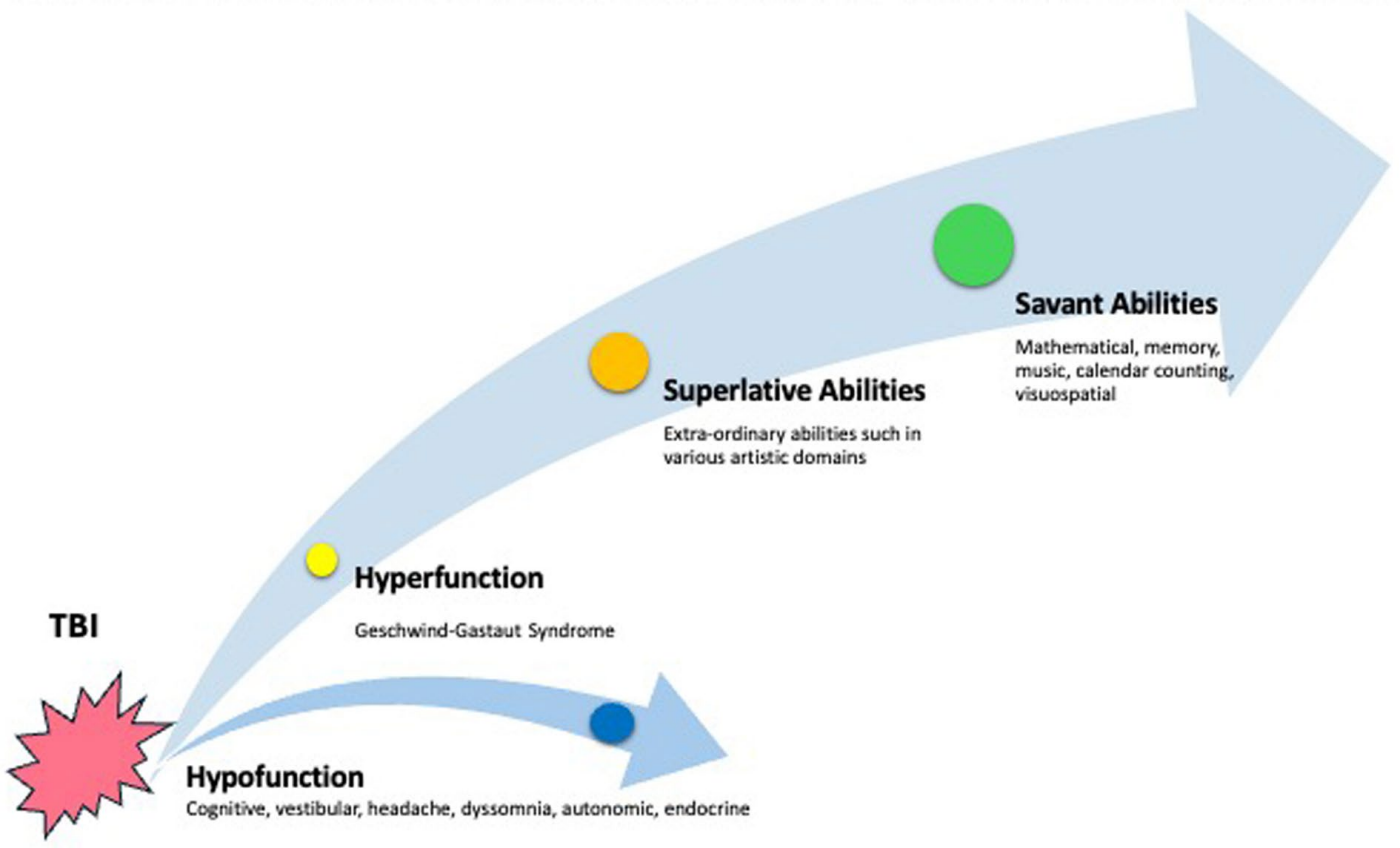
Traumatic brain injury: cerebral hypofunction, hyperfunction, superlative abilities and savant abilities.

Aside from the routine cognitive testing, administration of a battery of behavioral neurological tests, a number of common and less common syndromes may be identified. In this series, these included Diogenes syndrome, involuntary emotional expression disorders, attention deficit disorders, Klüver Bucy syndrome, and field-dependent behaviors, such as imitation behavior or utilization behavior. What may be less well known or appreciated is the number of enhanced cognitive, superlative, and rarely savant abilities that may emanate months or years later. Other than manifesting after the TBI, the time of onset of these abilities is difficult to determine and appears highly variable. These take the form of newly acquired artistry, musicality, language abilities, architectural abilities, as well as comedic expertise. Individuals who report newfound psychic abilities. The latter is not amenable to rigorous documentation, including one individual who reported the capability of clairvoyance. Psychic abilities post-TBI have been rarely reported to date (Gesen et al., 2021). The present study suggests that the range of behavioral neurological syndromes is much more common than has been appreciated to date, as also highlighted by Roy et al. (2023). The different syndromes that emerge after TBI, including the numerous hypofunction categories more commonly known as hyperfunction, superlative abilities, and rare savant capabilities, may be conceived as forming a continuum (Figure 2). The majority of the findings were diagnosed using structured instruments as delineated in Table 2, within the headings of common, less common, and uncommon disorders. Only the olfactory and visual syndromes, which are normally the case, and some of the superlative/hyperfunction syndromes have been diagnosed by participant self-reports (Table 2 sub-section superlative or hyperfunction syndromes). The latter pertains in particular to the single patient reporting clairvoyance abilities. Other than manifesting after the TBI, the time of onset of these abilities is difficult to determine and appears highly variable, and was noted to range from months to years and even decades in some.

**Figure 3:**
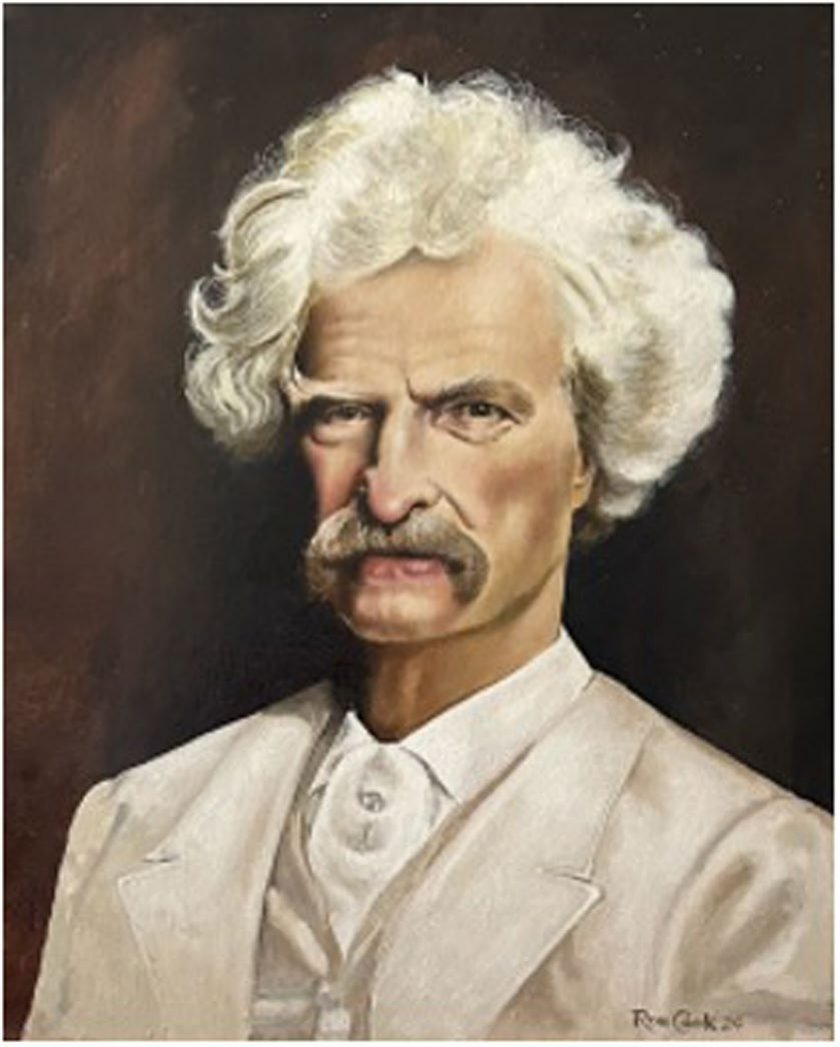
Mark Twain portrait rendition. With permission: RC.

The magnitude and spectacular examples that were documented by these individuals deserve separate reporting as an adjunct to this manuscript, but a sample artwork with signed consent by one individual is noted (Figure 3). This has been attributed to the known right hemisphere function of artistic, musical, and creative functions. Their identification is important in that these provide opportunities for a more precise understanding of the individuals’ poly-symptomatic presentations, management, and treatment options. For example, a case report of artistic excellence post-TBI was documented to facilitate improved activities of daily living in one individual, and a systematic review of arts engagement has been correlated with reducing cognitive decline as well as improving quality of life in otherwise healthy older people (Iacono and Feltis, 2025; Fioranelli et al., 2023). The information by participants who relayed impressive psychic abilities and savant-type abilities to the clinician was nevertheless regarded as unverified self-reports. Hence, the veracity of the cited abilities may remain in question. This did not apply to some of the superlative abilities, such as artistic brilliance, where factual evidence was provided, as in Figure 3 and published books and online publications by others, which can be accessed on file.

Research emanating from the stroke as well as the TBI literature has documented a number of different cerebral deactivations, remote from the offending lesion’s site (Mollica et al., 2025; Papini et al., 2024). Right and left hemisphere syndromes due to contralateral hemisphere damage, depicted by neuroimaging generally more clearly delineated in the stroke as opposed to the TBI literature (Thiebaut de Schotten et al., 2020). The range of diaschisis syndromes was eloquently presented by Carrera and Tononi (2014), including diaschisis at rest, functional diaschisis, connectional diaschisis, and connectomal diaschisis. Cerebral connectional diaschisis theories, in particular, may explain the syndromes reported in this study. Of particular relevance to TBI, the salience network with its unique neuronal VEN populations, some of which project widely to other parts of the cerebrum, is being optimized for rapid communications. Social behaviors and their coordination require such high-speed transmissions, providing the most important core essentials of human interaction (Bonnelle et al., 2012).

Different from stroke or other pathologies that are more focal, TBI may lead to either right or left hemisphere hyperactivation, as well as triggering large-scale network dysfunction. Efficient behavior is mediated by the coordinated activity of large-scale brain networks, with the salience network (SN) in particular being important for coordinating the activity of the other two major networks, the default mode network (DMN) and executive control network (ECN). The SN comprises the anterior cingulate cortex, anterior insulae, and presupplementary motor areas, and damage to the structural connectivity of the SN would lead to dysregulation of associated networks, as traumatic axonal injury leads to dysconnectivity of brain networks with disruption of white matter tracts (Sharp et al., 2014). DMN deactivation has been documented in TBI patients, attributed to an impairment of inhibitory control mechanisms, and DMN function correlated with the degree of white matter damage in the SN connections with the right anterior insula, dorsal anterior cingulate, and presupplementary motor area (Bonnelle et al., 2012). In general, a function of SN is in mediating rapid responses to perceived potential threats integrated with interoceptive information, as well as controlling the switching between the DMN and ECN networks (Uddin, 2015). Hence, the signature syndrome of TBI is the resulting disconnectome affecting widespread cerebral regions and networks. The authors offer the foregoing explanation as a biologically plausible and contemporary framework for interpreting the reported clinical observations (Reviewer 3). This also pertains to blast injuries that also manifested diffuse axonal injury, which causes shearing of neuronal synapses that may likely explain transient loss of consciousness, dizziness, and, in general, neuronal communication impairment (Przekwas et al., 2016). Even mild TBI without discernible exterior wounds, including blast injuries, is known to damage white matter tracts extensively throughout the neuraxis. In order of frequency, the most involved white matter tracts were; the corticospinal tract (88.2%), anterior thalamic radiations (81.2%), cingulum (60.0%), superior longitudinal fasciculi (55.9%), inferior longitudinal fasciculi (27.4%), uncinate (27.1%), and fornices (27.1%) with a correlation between white matter microstructural damage and neuropsychological symptoms, ascertained by diffusion tensor brain MRI, and cognitive testing (Beucler et al., 2025).

Overall, brain regions and networks affected specifically with TBI include the (i) orbitofrontal cortex and anterior temporal lobe (predilection due to anatomy), (ii) uncinate fasciculus (large local network), and (iii) the large-scale salience network interacting with other ICNs, specifically DMN, ECN, and semantic appraisal networks. The frontotemporal dementia literature has implicated the same network involvement and dysfunction. In behavioral variant FTD, the salience network in particular is regarded as most relevant in view of its mediating social–emotional-autonomic details (Restrepo-Martinez et al., 2024; Seeley et al., 2012). Also relevant to FTS, is the semantic appraisal network (SAN), also referred to as the limbic network, which has been correlated with emotion identification. The key hubs are in the anterior temporal lobe, rostral medial prefrontal cortex, subgenual anterior cingulate cortex, nucleus accumbens, and basolateral amygdala (Yand et al., 2021).

The importance of identifying the numerous subsyndromes, including cognitive, behavioral, neurological, elementary neurological, and neuropsychiatric subsyndromes, paves the way for a precision medicine-type treatment approach that allows targeted treatment and neurorehabilitation. Hence, a wide variety of clinicians may be the first port of call for the wide-ranging post-TBI syndromes, including primary care physicians, psychiatrists, psychologists, physical medicine and rehabilitation, and neurologists. Post mild TBI, WM integrity assessment may also benefit from additional blood-based biomarkers such as p-tau, in addition to diffusion MR imaging (Papini et al., 2024) and the more recently reviewed divided Subtracted Inversion Recovery (dSIR) sequences imaging that are most sensitive to minor white matter changes (Cornfeld et al., 2025). Such clinical, imaging, and blood-based testing helps pave the way for future treatments such as TMS, photo-biomodulation, acoustic stimulation, and Eye Movement Desensitization and Reprocessing (EMDR) therapy (de Voogd et al., 2018; Janssen et al., 2023). Contrary to previous teaching, recovery after mild TBI is not always rapid and at times may persist for months and even years. Pertinent in this regard are the core neural underpinnings of persisting symptoms after mild TBI, which have recently been identified as the salience network, the neural hub that is disproportionately involved. The DLPFC is also co-activated with the salience network and may be a potential candidate network for targeted neuromodulation (Mollica et al., 2025).

## Potential limitations and strengths of the review

A potential criticism of the study pertains to the flaws of retrospective studies, with limited causality attributed to the observational nature of such studies. In addition, selection bias with the inability to represent the broader population may be associated with the skewing of test results and outcomes. A notable limitation of this study is the lack of a comparison group and the accompanying inherent selection bias. In this study, the predominance of men, many of whom had complex conditions, and although TBI was present in the majority, such factors may predispose to selection bias and may not generalize to the broader TBI population. The small number of women represented (*n =* 9) precluded further gender analysis due to the predominance of combat-related veterans in this analysis.

As the wide variety of syndromes emanated, variously from symptom inventories, specific questionnaires, and clinical neurological assessments, unavoidable overlap is to be expected in the more frequent diagnoses of frontotemporal syndromes, executive dysfunction, disinhibition, and Geschwind-Gastaut syndrome. Participants in the study often had their heralding TBI many years and in some cases decades before their assessment. To the best of the investigators’ history taking and assessment, the syndromes encountered were related to the TBI, and no prior psychiatric or confounding conditions were documented, but this nevertheless remains uncertain.

Approaches used in scientific analysis include both a more traditional hypothesis-driven method and a data-driven approach. The former method uses specific predictions based primarily on proposed hypotheses and largely based on intuition. On the other hand, the data-driven approach utilizes extensive data accrued via registries or databases that enable the detection of specific patterns in conditions, syndromes, and diseases that inform a much more veritable hypothesis formulation. Hence, an advantage of a case series review is that it provides a screening tool for the most likely or plausible hypotheses that can then be targeted for further analysis. Furthermore, a specific advantage of the case is in generating new hypotheses and assessing potential treatment efficacy. Therefore, a potential strength of this retrospective case series includes high external validity, which frequently exceeds that of a randomized controlled trial; there was no interference in the treatment process, the study was inexpensive and short in duration (Talari and Goyal, 2020). This clinical registry can therefore infer hypotheses and guide lesion network associations and guide future studies to discern these complex syndromes with imaging-behavior associations with control groups, which the authors envisage for future studies.

Nevertheless, the findings of this study need to be cautiously generalized and interpreted as exploratory and as hypothesis-generating observations. The resultant frequencies of Geschwind-Gastaut-like features and frontotemporal characteristics, for example, need to be understood as a feature of the internal composition of the study as opposed to prevalence estimates pertaining to the broader traumatic brain injury population. The male predominance, predominantly being combat veterans, and no control group, may all influence the interpretation and generalizability of the study. These observed findings within a specific clinical cohort do not allow causal conclusions as direct comparisons with neurologically normal individuals were not possible. Until replicated by controlled comparative designs, these findings need to be interpreted with caution. This clinical registry can therefore infer hypotheses and guide lesion network associations and guide future studies to discern these complex syndromes with imaging-behavior associations with control groups, which the authors envisage for future studies.

## Conclusion

Guided by the commonality of post-TBI frontotemporal syndromes, a clinically useful approach is to deconstruct the overarching FTD diagnosis into multiple subsyndromes. This study noted both hypofunction syndromes and a notable proportion of both hyperfunction and superlative function categories. This study hopes to underscore the possibility of a range of neurological stealth syndromes as part of the post TBI range of maladies that facilitate a more targeted, precision management approach.

## Data Availability

The raw data supporting the conclusions of this article will be made available by the authors, without undue reservation.
