# Supplementary material for: Traumatic brain injury and neurological stealth syndromes
Source: Front Neurosci. 2026 Jul 15;20:1879688. doi: 10.3389/fnins.2026.1879688 (PMC13415762; doi:10.3389/fnins.2026.1879688)
Supplement: Supplementary file 1 [file Supplementary_file_1.pdf]

Appendix 1. Table A. Linear regression model of the associations between syndrome diagnosis and principal tests (number of patients n = 73).

| | FRS IEED | FRSB ADHD | FBI Diogenes | DMIS | FBI IM/UB | GGS |
|---|---|---|---|---|---|---|
| Female | -0.137 | -0.166 | 0.257 | 0.012 | 0.094 | 0.022 |
| | (-0.452, 0.179) | (-0.469, 0.137) | (-0.113, 0.627) | (-0.082, 0.107) | (-0.159, 0.348) | (-0.234, 0.279) |
| Education (years) | 0.023 | -0.021 | -0.039 | -0.001 | 0.023 | -0.014 |
| | (-0.027, 0.073) | (-0.069, 0.027) | (-0.097, 0.020) | (-0.015, 0.014) | (-0.017, 0.063) | (-0.055, 0.026) |
| Age of patient | -0.002 | 0.010* | 0.010 | -0.001 | -0.003 | -0.003 |
| | (-0.011, 0.006) | (0.002, 0.018) | (0.000, 0.020) | (-0.003, 0.002) | (-0.010, 0.004) | (-0.010, 0.004) |
| Body Mass Index | 0.015 | 0.001 | 0.003 | 0.004 | 0.020* | 0.002 |
| | (-0.006, 0.036) | (-0.019, 0.021) | (-0.021, 0.027) | (-0.002, 0.010) | (0.004, 0.037) | (-0.015, 0.019) |
| MOCA score | 0.008 | -0.006 | 0.026 | -0.004 | 0.024 | 0.003 |
| | (-0.025, 0.041) | (-0.038, 0.025) | (-0.013, 0.065) | (-0.013, 0.006) | (-0.002, 0.051) | (-0.024, 0.029) |
| Boston Naming test | -0.011 | -0.012 | -0.046 | 0.009 | -0.062* | 0.055* |
| | (-0.077, 0.055) | (-0.075, 0.051) | (-0.123, 0.031) | (-0.011, 0.028) | (-0.115, -0.010) | (0.001, 0.108) |
| FBI score | 0.001 | 0.014* | 0.011 | 0.000 | -0.003 | -0.013* |
| | (-0.013, 0.014) | (0.001, 0.027) | (-0.004, 0.026) | (-0.004, 0.004) | (-0.013, 0.008) | (-0.024, -0.002) |
| FRSBE A | -0.001 | 0.008 | 0.006 | -0.002 | 0.000 | -0.002 |
| | (-0.012, 0.011) | (-0.003, 0.019) | (-0.007, 0.019) | (-0.005, 0.002) | (-0.009, 0.009) | (-0.011, 0.007) |
| FRSBE D | 0.018** | 0.023** | 0.006 | -0.001 | 0.004 | 0.004 |
| | (0.005, 0.031) | (0.010, 0.035) | (-0.009, 0.021) | (-0.005, 0.003) | (-0.006, 0.014) | (-0.007, 0.014) |
| FRSBE E | 0.005 | 0.016 | 0.002 | -0.003 | -0.011 | 0.003 |
| | (-0.018, 0.027) | (-0.005, 0.037) | (-0.024, 0.028) | (-0.010, 0.004) | (-0.029, 0.007) | (-0.015, 0.021) |
| FRSBE T | -0.007 | -0.039* | -0.010 | 0.006 | 0.012 | 0.001 |
| | (-0.040, 0.027) | (-0.071, -0.007) | (-0.049, 0.029) | (-0.004, 0.016) | (-0.015, 0.038) | (-0.026, 0.028) |
| Bear Fedio | 0.008 | 0.021 | 0.003 | 0.002 | -0.011 | 0.088** |
| | (-0.022, 0.038) | (-0.007, 0.050) | (-0.032, 0.038) | (-0.007, 0.011) | (-0.035, 0.013) | (0.064, 0.113) |
| MRI Brain | 0.112 | -0.261 | -0.059 | -0.006 | 0.021 | -0.081 |
| | (-0.209, 0.432) | (-0.569, 0.047) | (-0.435, 0.316) | (-0.102, 0.089) | (-0.237, 0.278) | (-0.342, 0.179) |
| Daphne FTD | 0.090 | 0.080 | -0.199 | -0.141** | -0.165 | -0.124 |
| | (-0.226, 0.405) | (-0.224, 0.383) | (-0.569, 0.172) | (-0.235, -0.047) | (-0.418, 0.089) | (-0.381, 0.133) |

** p<0.01, * p<0.05

| | KBS | Hypergraphia | Circumstantiality | Viscosity | Emotionality | Hypermoralism |
|---|---|---|---|---|---|---|
| Female | -0.042 | -0.183 | -0.035 | 0.276 | 0.014 | 0.133 |
| | (-0.331, 0.248) | (-0.490, 0.124) | (-0.417, 0.346) | (-0.063, 0.615) | (-0.345, 0.373) | (-0.181, 0.447) |
| Education (years) | -0.014 | 0.017 | -0.011 | 0.000 | 0.023 | 0.005 |
| | (-0.060, 0.032) | (-0.032, 0.066) | (-0.072, 0.049) | (-0.053, 0.054) | (-0.034, 0.080) | (-0.045, 0.055) |
| Age of patient | -0.005 | 0.001 | -0.002 | -0.001 | 0.001 | -0.001 |
| | (-0.013, 0.003) | (-0.007, 0.009) | (-0.013, 0.008) | (-0.010, 0.008) | (-0.009, 0.010) | (-0.009, 0.008) |
| Body Mass Index | 0.010 | -0.005 | 0.001 | 0.002 | -0.002 | 0.009 |
| | (-0.009, 0.029) | (-0.025, 0.016) | (-0.024, 0.026) | (-0.020, 0.024) | (-0.026, 0.021) | (-0.011, 0.030) |
| MOCA score | -0.005 | 0.028 | -0.010 | 0.023 | -0.008 | -0.025 |
| | (-0.035, 0.026) | (-0.004, 0.060) | (-0.050, 0.029) | (-0.013, 0.058) | (-0.046, 0.029) | (-0.057, 0.008) |
| Boston Naming test | -0.030 | -0.031 | 0.012 | -0.009 | -0.025 | 0.076* |
| | (-0.091, 0.030) | (-0.095, 0.033) | (-0.068, 0.091) | (-0.080, 0.061) | (-0.099, 0.050) | (0.011, 0.142) |
| FBI score | 0.008 | -0.004 | -0.004 | -0.016* | -0.010 | 0.011 |
| | (-0.005, 0.020) | (-0.017, 0.009) | (-0.020, 0.012) | (-0.030, -0.002) | (-0.025, 0.005) | (-0.002, 0.025) |
| FRSBE A | -0.006 | -0.009 | 0.004 | -0.007 | 0.011 | -0.006 |
| | (-0.016, 0.005) | (-0.020, 0.002) | (-0.010, 0.018) | (-0.020, 0.005) | (-0.002, 0.024) | (-0.017, 0.006) |
| FRSBE D | -0.004 | -0.009 | 0.001 | -0.009 | 0.016* | -0.001 |
| | (-0.016, 0.008) | (-0.021, 0.004) | (-0.015, 0.016) | (-0.023, 0.005) | (0.001, 0.031) | (-0.013, 0.012) |
| FRSBE E | 0.002 | -0.015 | 0.007 | -0.002 | 0.014 | -0.004 |
| | (-0.019, 0.022) | (-0.037, 0.006) | (-0.019, 0.034) | (-0.025, 0.022) | (-0.011, 0.039) | (-0.026, 0.018) |
| FRSBE T | 0.008 | 0.031 | -0.009 | 0.020 | -0.035 | 0.009 |
| | (-0.022, 0.039) | (-0.001, 0.063) | (-0.049, 0.031) | (-0.016, 0.056) | (-0.073, 0.003) | (-0.024, 0.042) |
| Bear Fedio | 0.016 | 0.055** | 0.073** | 0.075** | 0.068** | 0.033* |
| | (-0.011, 0.043) | (0.026, 0.084) | (0.036, 0.109) | (0.043, 0.107) | (0.034, 0.102) | (0.003, 0.063) |
| MRI Brain | 0.070 | -0.097 | -0.001 | -0.055 | 0.012 | -0.094 |
| | (-0.224, 0.364) | (-0.409, 0.215) | (-0.388, 0.386) | (-0.399, 0.289) | (-0.352, 0.376) | (-0.413, 0.225) |
| Daphne FTD | -0.070 | -0.128 | 0.096 | -0.369* | -0.007 | 0.232 |
| | (-0.359, 0.220) | (-0.436, 0.179) | (-0.286, 0.478) | (-0.708, -0.030) | (-0.366, 0.352) | (-0.082, 0.547) |

** p<0.01, * p<0.05

| | Obsession | Personal destiny | Philosophical | Hyper religious | Sexual alteration | Depression |
|---|---|---|---|---|---|---|
| Female | 0.121 | 0.087 | -0.391* | -0.014 | -0.431* | -0.118 |
| | (-0.206, 0.447) | (-0.270, 0.444) | (-0.700, -0.083) | (-0.305, 0.276) | (-0.782, -0.079) | (-0.451, 0.215) |
| Education (years) | 0.058* | 0.008 | -0.023 | -0.041 | -0.009 | -0.047 |
| | (0.006, 0.109) | (-0.049, 0.064) | (-0.071, 0.026) | (-0.087, 0.005) | (-0.065, 0.046) | (-0.100, 0.006) |
| Age of patient | -0.009* | 0.001 | -0.002 | 0.010* | 0.002 | 0.003 |
| | (-0.018, -0.001) | (-0.008, 0.011) | (-0.010, 0.006) | (0.002, 0.018) | (-0.008, 0.011) | (-0.006, 0.012) |
| Body Mass Index | -0.012 | 0.008 | 0.000 | 0.013 | -0.001 | 0.003 |
| | (-0.034, 0.009) | (-0.016, 0.031) | (-0.020, 0.020) | (-0.006, 0.032) | (-0.024, 0.022) | (-0.019, 0.024) |
| MOCA score | -0.002 | 0.037 | 0.024 | 0.051** | 0.012 | -0.003 |
| | (-0.037, 0.032) | (0.000, 0.074) | (-0.008, 0.056) | (0.020, 0.081) | (-0.025, 0.049) | (-0.038, 0.032) |
| Boston Naming test | -0.020 | -0.039 | -0.016 | -0.014 | 0.009 | -0.002 |
| | (-0.088, 0.048) | (-0.113, 0.036) | (-0.080, 0.048) | (-0.075, 0.046) | (-0.064, 0.083) | (-0.071, 0.067) |
| FBI score | -0.006 | -0.010 | 0.002 | -0.012 | -0.006 | 0.000 |
| | (-0.019, 0.008) | (-0.025, 0.005) | (-0.010, 0.015) | (-0.024, 0.000) | (-0.021, 0.008) | (-0.014, 0.014) |
| FRSBE A | -0.008 | -0.004 | -0.003 | 0.004 | 0.010 | 0.012* |
| | (-0.020, 0.004) | (-0.017, 0.008) | (-0.015, 0.008) | (-0.006, 0.015) | (-0.003, 0.022) | (0.000, 0.024) |
| FRSBE D | -0.002 | 0.004 | -0.002 | 0.006 | -0.001 | 0.016* |
| | (-0.016, 0.011) | (-0.010, 0.019) | (-0.015, 0.010) | (-0.006, 0.018) | (-0.015, 0.014) | (0.002, 0.029) |
| FRSBE E | -0.014 | 0.008 | 0.007 | 0.020 | -0.001 | 0.015 |
| | (-0.036, 0.009) | (-0.017, 0.033) | (-0.015, 0.028) | (0.000, 0.040) | (-0.026, 0.023) | (-0.008, 0.039) |
| FRSBE T | 0.024 | -0.003 | 0.006 | -0.017 | -0.007 | -0.038* |
| | (-0.011, 0.058) | (-0.040, 0.035) | (-0.027, 0.038) | (-0.048, 0.013) | (-0.044, 0.030) | (-0.073, -0.003) |
| Bear Fedio | 0.071** | 0.043* | 0.063** | 0.038** | 0.079** | 0.011 |
| | (0.040, 0.102) | (0.010, 0.077) | (0.034, 0.092) | (0.010, 0.065) | (0.045, 0.112) | (-0.021, 0.042) |
| MRI Brain | 0.027 | 0.081 | 0.028 | 0.193 | -0.023 | 0.153 |
| | (-0.304, 0.359) | (-0.281, 0.444) | (-0.285, 0.341) | (-0.102, 0.488) | (-0.381, 0.334) | (-0.185, 0.492) |
| Daphne FTD | -0.140 | 0.045 | 0.104 | -0.355* | 0.331 | 0.282 |
| | (-0.467, 0.187) | (-0.313, 0.402) | (-0.205, 0.413) | (-0.646, -0.065) | (-0.022, 0.683) | (-0.052, 0.615) |

** p<0.01, * p<0.05

| | Anxiety | PTSD | Migraine |
|---|---|---|---|
| Female | -0.381* | -0.415* | -0.125 |
| | (-0.708, -0.054) | (-0.810, -0.020) | (-0.473, 0.223) |
| Education (years) | -0.080** | -0.034 | -0.019 |
| | (-0.132, -0.028) | (-0.097, 0.028) | (-0.074, 0.037) |
| Age of patient | -0.002 | -0.006 | -0.006 |
| | (-0.011, 0.007) | (-0.017, 0.005) | (-0.016, 0.003) |
| Body Mass Index | 0.003 | 0.006 | -0.004 |
| | (-0.018, 0.024) | (-0.020, 0.032) | (-0.027, 0.018) |
| MOCA score | -0.009 | 0.019 | -0.005 |
| | (-0.043, 0.025) | (-0.022, 0.061) | (-0.041, 0.032) |
| Boston Naming test | 0.035 | -0.023 | 0.070 |
| | (-0.033, 0.103) | (-0.105, 0.059) | (-0.003, 0.142) |
| FBI score | -0.001 | -0.006 | 0.003 |
| | (-0.015, 0.013) | (-0.022, 0.011) | (-0.012, 0.017) |
| FRSBE A | 0.016* | 0.010 | 0.013 |
| | (0.004, 0.028) | (-0.004, 0.024) | (0.000, 0.025) |
| FRSBE D | 0.024** | 0.015 | 0.019* |
| | (0.011, 0.038) | (-0.001, 0.031) | (0.005, 0.033) |
| FRSBE E | 0.028* | 0.017 | 0.033** |
| | (0.005, 0.051) | (-0.011, 0.044) | (0.009, 0.058) |
| FRSBE T | -0.058** | -0.032 | -0.054** |
| | (-0.092, -0.023) | (-0.074, 0.009) | (-0.090, -0.017) |
| Bear Fedio | 0.015 | -0.008 | 0.006 |
| | (-0.016, 0.046) | (-0.046, 0.029) | (-0.027, 0.039) |
| MRI Brain | 0.038 | 0.299 | 0.062 |
| | (-0.294, 0.370) | (-0.102, 0.700) | (-0.291, 0.415) |
| Daphne FTD | 0.243 | 0.210 | 0.181 |
| | (-0.084, 0.571) | (-0.185, 0.605) | (-0.167, 0.530) |

** p<0.01, * p<0.05.
