# Supplementary material for: Traumatic brain injury and neurological stealth syndromes
Source: Front Neurosci. 2026 Jul 15;20:1879688. doi: 10.3389/fnins.2026.1879688 (PMC13415762; doi:10.3389/fnins.2026.1879688)
Supplement: Supplementary file 2 [file Supplementary_file_2.pdf]

Appendix 2. Case definitions for syndromes and exclusion rules, diagnosed by a cognitive neurologist

FTS/FTD syndromes diagnosis

1. By the DAPHNE-6 screening evaluation, which required 4 or more of the 6 criteria to be positive, including disinhibition, apathy, lack of empathy, perseverations, hyperorality, and personal neglect
2. FTD syndromes were further delineated into five clinical 5 subtypes, including
    a. Behavioral
    b. Semantic aphasia
    c. Progressive non-fluent aphasia
    d. Corticobasal ganglionic syndrome
    e. Progressive supranuclear palsy
    f. FTD association with amyotrophic sclerosis

Geschwind-Gastaut Syndrome diagnosis required 4 of the 5 criteria to be positive

1. Viscous personality: considered to be the central component of GGS, which includes circumstantiality, interpersonal viscosity, tendency to repetition, prolongation of personal encounters, loquacious, hyper-narrative, overinclusive verbal discourse, excessive detail of information, and pedantic behavior
2. Hypergraphia: excessive writing, including diaries, autobiographies, and random notes
3. Intensified mental life: deepening of emotions, hyper-moralism, sense of personal destiny, philosophical with nascent metaphysical or moral speculations, cosmological theories, dependence, at the hands of fate, cosmic helplessness
4. Religiosity: multiple conversions, deep religious beliefs, mystical states
5. Altered sexual interest: hyposexuality, hypersexuality, gender dysphoria, transvestism

The Human Klüver Bucy syndrome diagnosis required a minimum of 3 components of the following

1. Visual agnosia
2. Loss of anger or fear responses with placidity or flattened affect
3. Altered sexual activity or orientation
4. Hyperorality, bulimia, or insatiable appetite
5. Hypermetamorphosis, which refers to a compulsion to manipulate objects in the immediate environment, also termed utilization behavior

Delusional misidentification syndrome (DMIS)

These syndromes were diagnosed by the following criteria, whereby a person incorrectly identifies or duplicates persons, places, objects, or even events, which may be learned by self-report or substantiated by family members or friends. Although over a dozen different DMIS syndromes have been described, only the 3 of the more common ones were included for analysis.

1. Capgras syndrome, which is the belief by the person that a familiar individual or even the person themselves has been replaced by an imposter (hypo-identification)

2. Fregoli's syndrome, which is the belief that an individual familiar to the person is actually impersonating and presenting themselves as a stranger (hyper-identification)
3. Intermetamorphosis, which refers to two people, both familiar to the person, who have interchanged identities with one another.

Involuntary emotional expression disorder (IEED) diagnosis

This syndrome diagnosis was made using items 2 and 6 of the FRSBE test questionnaire, which were used and were scored ≥3 on the 5-point Likert scale. This question delineated a syndrome characterized by spontaneous outbursts of crying, laughing, or both, occurring contextually inappropriately. These questions read as:

1. Item 2: "I am easily angered or irritated; I may have emotional outbursts for no apparent reason."
2. Item 6: "I laugh or cry too easily".

Diogenes syndrome (personal neglect) diagnosis

This syndrome diagnosis was made if item number 11 of the FRSBE test questionnaire, which scored ≥3 on the 5-point Likert scale, or if item 6 of the Frontal Behavioral Inventory (FBI) test was rated a 2 or 3 on a scale of 0-3
These questions read as:
Item 11 of the FRSBE: "Neglect my personal hygiene"
Item 6 of the FBI test: "Does he/she take as much care as usual of his/her personal hygiene and appearance as usual

Field-dependent behavior (FDB): Environmental autonomy (imitation and utilization behavior).

A diagnosis of imitation behavior was evaluated; while maintaining eye contact, the examiner pats the side of the face and then claps the hands without suggesting the patient follow suit. The diagnosis of utilization behavior: Place three objects in front of the patient: a key, a cell phone, and a pen. UB was also diagnosed if item 22 of the FBI test was ≥2 on a scale of 0-3. Does s/he seem to need to touch, feel, examine, or pick up objects within reach and sight [19].

1. A diagnosis of imitation behavior was made if the person copies the action without being asked to do so; this was scored as positive
2. A diagnosis of utilization behavior was made if the person manipulates or uses the objects in any way; this is scored as positive.
3. A diagnosis of utilization behavior was also made if item 22 of the FBI test was rated a 2 or 3 on a scale of 0-3

Attention-deficit/hyperactivity diagnosis

This syndrome diagnosis was made if item numbers 4 and 12 of the FRSBE test scored ≥3 on the 5-point Likert scale, which was used as a positive diagnosis, as well as by DSM-5 criteria [31]

1. Item 4: "I do things impulsively"
2. Item 12: "Can't sit still, am hyperactive"

Depression

This diagnosis was made in accordance with the DSM-5 criteria [31].

Anxiety

This diagnosis was made in accordance with the DSM-5 criteria [31]

Post-traumatic stress disorder (PTSD)

This diagnosis was made in accordance with DSM-5 criteria [31]

Traumatic Brain Injury diagnosis

This diagnosis was made in accordance with the Centers for Disease Control and ICD-10 criteria for mild and moderate TBI (33).

Frontotemporal lobe generic diagnosis, including degenerations and dementias [17,34]

Screening test for frontotemporal disorders with the Frontal Behavioral Inventory (FBI test), with a positive diagnosis made if the score is ≥24 [18].
